# A Scoping Review of Eosinophilic Pneumonia and Antidepressants: An Association Not to Be Overlooked

**DOI:** 10.3390/diseases13010013

**Published:** 2025-01-13

**Authors:** Jaron Steiner, Leonie Steuernagel, Fotios Drakopanagiotakis, Konstantinos Bonelis, Paschalis Steiropoulos

**Affiliations:** 1 Charité University Hospital, 10117 Berlin, Germany; jaron-ben-michael.steiner@charite.de (J.S.); leonie-anabell.steuernagel@charite.de (L.S.); 2 Department of Pneumonology, Medical School, Democritus University of Thrace, University General Hospital Dragana, 68100 Alexandroupolis, Greece; kon.bonelis@gmail.com (K.B.); steiropoulos@yahoo.com (P.S.)

**Keywords:** eosinophilic pneumonia, antidepressants, SSRI, acute eosinophilic pneumonia, chronic eosinophilic pneumonia

## Abstract

*Background*: Eosinophilic pneumonias denote a rare condition, wherein infiltrating eosinophilic granulocytes accumulate within the lung parenchyma. Although eosinophilic pneumonias may be idiopathic, they are also associated with secondary causes. More than 110 medications have been linked to eosinophilic pneumonia, including several antidepressants. This review presents an analysis of case reports of eosinophilic pneumonia correlated to antidepressants. *Objectives*: The objectives of this study are to provide a contemporary overview of the literature delineating eosinophilic pneumonia as a potential sequela of antidepressant medication treatment, and to discuss possible pathogenetic mechanisms linking antidepressants to eosinophilic pneumonia. *Methods and Data Selection*: A literature search was performed in PubMed and Scopus databases from 1963 to October 2024. The search strategy used the terms “eosinophilic pneumonia AND antidepressants”. Sources included in this review were screened for relevance, focusing on references discussing eosinophilic pneumonia associated with any class of antidepressants. Case reports meeting the diagnostic criteria for acute eosinophilic pneumonia (AEP) or chronic eosinophilic pneumonia (CEP) were included in the review. Clinical, epidemiological, laboratory, radiology and bronchoscopy data, implicated antidepressant and dosage, and therapeutic interventions were reported. *Results*: This study found that various types of antidepressants are associated with AEP and CEP. The clinical presentation ranges from mild symptoms to respiratory failure and intubation. Outcomes were favorable in most cases, with complete remission achieved after discontinuation of the causative drug and, in severe cases, a short course of corticosteroids. *Conclusions*: Although a rare cause, antidepressants may lead to eosinophilic pneumonia, and should be considered in the differential diagnosis of unexplained pulmonary infiltrates. Clinical suspicion must be aroused, as early recognition would prevent unnecessary work-up and navigation of the diagnosis.

## 1. Introduction

Eosinophilic pneumonias are a rare syndrome characterized by infiltrating eosinophilic granulocytes accumulating within the lung parenchyma. This accumulation is primarily seen in the alveoli, but the interstitium may also be affected [1]. Despite the eosinophilic inflammation, the typical lung architecture remains intact. Although lymphocytes and neutrophils may be present in the lung parenchyma and interstitium, these cell types do not predominate. Peripheral blood eosinophilia is present in approximately 80% of cases, along with consistent eosinophilia (>25% eosinophils) in bronchoalveolar lavage (BAL) [2]. The classification of eosinophilic pneumonia is primarily divided between unknown and known causes [1,2,3]. This classification is shown in Table 1.

More than 210 medications have been linked to eosinophilic pneumonias, which encompass a broad spectrum of manifestations [4]. These range from transient pulmonary infiltrates, as in the case of Löffler syndrome, to acute and chronic eosinophilic pneumonia, eosinophilic granulomatosis with polyangiitis (EGPA, formerly known as Churg–Strauss Syndrome), or hypereosinophilic syndrome [1,2,3]. After discontinuation of the medication, the disease usually regresses quickly, although treatment with systemic corticosteroids may be necessary depending on the severity and progression of the eosinophilic disease [5]. A severe, acute drug reaction associated with skin rash and visceral organ involvement is the drug reaction with eosinophilia syndrome (DRESS syndrome) [4,6]. Patients with DRESS syndrome may experience symptom relapses despite drug discontinuation [4,6].

Antidepressants are used clinically to treat patients suffering from major depression disorders and anxiety disorders [7]. Antidepressants have also been associated with the development of eosinophilic pneumonia, particularly with chronic and acute eosinophilic pneumonia phenotypes. In this scoping review, we discuss the clinical characteristics of acute and chronic eosinophilic pneumonias, the pathophysiology of eosinophilic pneumonias, and assess the available evidence regarding the association between antidepressants and eosinophilic pneumonia. Furthermore, we provide a summary of these studies’ key clinical and radiological results.

### 1.1. Acute Eosinophilic Pneumonia

Acute eosinophilic pneumonia (AEP) is a rare acute lung inflammation, first described in 1989 [8]. The disease may be idiopathic or may be associated with specific triggers. Such triggers include medications, the most known being the ones correlated to antibiotics. Nitrofurantoin, minocycline, and daptomycin are some common examples [2]. AEP has also been described as a side effect of antidepressants [4]. *Sertraline, clomipramine, desipramine, venlafaxine, duloxetine, amitryptiline, and vortioxetine have been associated with* AEP [4]. 

AEP is an acute onset condition that predominantly affects young men (10:1 ratio) around 25 years of age (range of 15 to 86 years), and often leads to severe hypoxemia. Cigarette smoke (90%) appears to be a significant triggering factor in the idiopathic form [2]. Other possible causes include various forms of inhalation, smoking (e-cigarettes, marijuana, crack, heroin), smoke, gas, or dust exposure (e.g., desert dust, World Trade Center dust, or even allogeneic stem cell transplantation) [2].

The pathogenesis is unclear, but AEP often occurs shortly after a rapid onset of smoking, and some patients experience recurrence after re-exposure to cigarette smoke. Diagnosis requires bronchoalveolar lavage to confirm the increased eosinophil count. Additional requirements are the exclusion of known causes of pulmonary eosinophilia, such as medications, infections, and allergies. Clinically, radiologically accompanied by BAL cytology (>25% eosinophils), drug-induced disease patterns do not differ from idiopathic and other forms of pulmonary eosinophilia [2,5].

Eosinophilic pneumonia typically presents with an acute onset and rapidly progressive course, leading to severe respiratory failure in less than four weeks, often within less than one week. The predominant symptoms include dyspnea and cough, which are present in approximately 95% of patients, alongside a fever of around 39 °C, observed in about 90% of the affected individuals [2,3,5].

The diagnosis of acute eosinophilic pneumonia is based on specific criteria. These include an acute onset with symptoms such as cough, dyspnea, and fever lasting less than one month, bilateral diffuse infiltrates, severe hypoxemia, and eosinophilia exceeding 25% in the bronchoalveolar lavage (BAL) fluid, with or without blood eosinophilia [2,3,5]. In cases of AEP associated with antidepressants, a temporal correlation of the symptoms and radiologic findings to the start of treatment with the antidepressant and its resolution after treatment discontinuation are essential diagnostic criteria [9,10,11].

### 1.2. Chronic Eosinophilic Pneumonia

Chronic eosinophilic pneumonia (CEP) is a rare, chronic lung inflammation characterized by peripheral and pulmonary eosinophilia. The disease is defined as idiopathic when an identifiable cause cannot be found, and when other organs are not involved [2,3,5]. CEP affects predominantly women (ratio 2:1), with 30 to 50% of cases having a history of asthma, and about 60% having atopy. Notably, 90% of those affected are non-smokers [2].

The onset of CEP is insidious, presenting with constitutional symptoms such as fatigue, fever, weight loss, and night sweats, alongside respiratory symptoms, with cough (most commonly productive) and dyspnea in up to 90% of cases. Blood tests typically reveal severe blood eosinophilia (>1000 cells/μL) [2].

The diagnostic criteria include typical clinical and radiological features (predominately peripheral, subpleural, mid-to-upper zone opacities) accompanied with excessive BAL eosinophilia ≥40% (minimum 25%). Most commonly, peripheral blood eosinophilia is present with an absolute count of ≥1 ×  10^9^ cells/L. Respiratory symptoms persisting or worsening for 1 to 4 months, with no evidence of other organ involvement, diffuse ground glass opacities, or consolidations with air bronchograms, primarily in mid-upper and peripheral regions, are common findings. Additionally, idiopathic chronic eosinophilic pneumonia is characterized by the exclusion of known causes of eosinophilic lung disease and a rapid response to corticosteroids [1,2,5].

As in the case of AEP, CEP has been associated with several medications, particularly antibiotics and NSAIDS [4]. Antidepressants, including *sertraline* [12], *desipramine* [13], *maprotiline* [14], *duloxetine* [15], and *trimipramine* [16] have been reported as causes of CEP.

### 1.3. The Pathophysiology of Eosinophilic Granulocytes

Eosinophils were first identified and described by Elie Metchnikoff, followed by Paul Elhrich’s observation of their eosin-staining acidophilic granules. Eosinophils contribute significantly to the host defense against parasites (mainly helminths), and in pathological roles such as tissue damage, inflammation, remodeling, immune-mediated diseases, and allergic reactions [17].

Mature eosinophilic granulocytes comprise the final differentiation product of eosinophil progenitor cells (EoP) in bone marrow. EoP are derived from Granulocyte Macrophage Progenitor (GMP), a common progenitor of all granulocytes, expressing interleukin 5 receptors (IL-5R) [17]. Eosinophilic granulocytes undergo proliferation and terminal differentiation in the bone marrow under the influence of various cytokines and interleukins, particularly IL-3, IL-4, IL-5, and granulocyte-macrophage colony-stimulating factor (GM-CSF) [18]. This differentiation process is primarily regulated by T-lymphocytes, especially the T-helper (Th) type 2 lymphocytes, accompanied by Innate Lymphoid Cells (ILC2), mast cells, and macrophages [17,18]. New therapies targeting IL-5 or the specific receptor of this cytokine, IL-5R, have been proven to deplete eosinophils from blood, tissues, and bone marrow. However, complete depletion of eosinophils is not achieved. Alternative pathways of continuous differentiation of eosinophilic granulocytes occur via autocrine expression and signaling by anti-apoptotic survival factor GM-CSF, along with IL-3- and eosinophil-recruiting chemokines (CCL11, CCL24, CCL26) [17,18]. In healthy individuals, for homeostatic purposes, eosinophil development and maturation include a combination of transcription factors, with GATA-1 having a crucial role [17,18].

After maturation, eosinophils typically spend approximately one day in circulation before migrating into tissues. Less than 1% of the total number of eosinophils in the body is found in blood. Most of them are recruited in tissues, mainly the mucosa of the digestive tract, but also in the lung, thymus, uterus, mammary gland, and adipose tissue [17]. This recruitment is facilitated by chemotaxis, adhesion, and diapedesis processes, primarily regulated by IL-5 and eotaxin-1 (CCL11) [17,18]. Upon migration to tissue, the eosinophils accumulate and release numerous toxic substances from their secondary granules, notably the following four cationic proteins: major basic protein 1 (MBP1; also known as MBP and PRG2), eosinophil cationic protein (ECP; also known as RNase3), eosinophil-derived neurotoxin (EDN; also known as RNase2), and eosinophil peroxidase (EPX; also known as EPO). Within tissues, eosinophils undergo apoptosis unless there are survival factors that promote their proliferation [3,17,18].

Although eosinophils play a significant role in host defense, tissue regeneration, and tissue repair after injury [2], the excessive tissue accumulation of activated eosinophils can result in tissue damage [2]. Tissue accumulation of eosinophils is not always associated with the levels of eosinophils in blood, although blood levels above 1500/µL are most commonly related to subsequent tissue damage [17]. An increase in eosinophils in tissues or blood may be a result of polyclonal expansion due to increased IL-5 production, as seen in allergen exposure, helminthic infection, or EGPA [17]. A monoclonal expansion can be seen in hematologic malignancies, such as eosinophilic leukemia or hypereosinophilic syndrome [2,17,19]. Eosinophilic pneumonias may be a manifestation of a broad spectrum of eosinophilic lung diseases, affecting the airways in mild forms and extending to eosinophilic vasculitis [2]. This is supported by the observation that up to 75% of patients with CEP have asthma [2].

In AEP, the pathogenesis is largely unknown. It is assumed that idiopathic AEP represents an acute hypersensitivity reaction to an unknown antigen. Inhaled antigens, such as those contained in tobacco smoke or vaping, may act as triggers. Epithelial injury leads to Th2-cell activation and eosinophil recruitment in the lungs [20]. The pathologic correlate of AEP is diffuse alveolar damage with infiltration of the airspaces, interstitium, and bronchioles by eosinophils [2]. Regarding the association of AEP with antidepressants, it seems that serotonin 2A (5-HT2A) acts as an inflammatory mediator, regulating cytokine release in airway epithelial cells. Moreover, serotonin has a chemoattractant action for eosinophils [5].

CEP, on the other hand, may be idiopathic or associated with various causes, such as drugs. CEP is characterized by extensive infiltration of the alveolar airspaces and the interstitium by eosinophils. The lung architecture is preserved, a finding similar to organizing pneumonia [2]. Cytotoxic injury and immune-mediated injury in predisposed individuals have been proposed as possible mechanisms of drug-induced CEP [5]. Regarding antidepressants, serotonin induces a pro-inflammatory response and influences the priming capacity of dendritic cells on T-cells, favoring a Th2-cell profile. This action leads to eosinophilic recruitment in allergic airway diseases [5,21].

### 1.4. Classification of Antidepressants

Antidepressants are presented based on the ATC and NbN classification [22,23]. The substances of the first generation of antidepressants, tricyclic antidepressants (TCA) (antidepressants of the first generation), are trimipramine, desipramine, imipramine, clomipramine, and amitriptyline. Maprotiline is a tetracyclic antidepressant (TeCA) [24,25].

Second-generation antidepressants can be divided into two different classes, as follows: selective serotonin reuptake inhibitors (SSRIs) and other second-generation antidepressants. Among the SSRIs, fluoxetine, sertraline, and *paroxetine* have been reported to cause eosinophilic pneumonia. Among the serotonin and noradrenaline reuptake inhibitors (SNRIs), *duloxetine* and *venlafaxine* were identified as causes of eosinophilic pneumonia. Noradrenaline and dopamine reuptake inhibitors (NDRIs) and the new multimodal antidepressants all fall under the heterogeneous class of second-generation antidepressants. Representers of this class are *trazodone*, a serotonin antagonist and reuptake inhibitor (SARI), and *vortioxetine*, a serotonin modulator and stimulator (SMS)*,* which were reported to cause eosinophilic pneumonia [26].

Generally, antidepressants modify concentrations of noradrenalin and serotonin. Different mechanisms lead to raised levels of noradrenalin and serotonin in the synaptic cleft. TCAs originate from tricyclic neuroleptics and target the inhibition of 5-HT2A-/5-HT2C- and H1- receptors, as well as the reuptake of noradrenaline and serotonin, while being relevantly metabolized through CYP2D6. SSRIs mainly inhibit serotonin transport from the synaptic cleft by inhibiting the serotonin transporter [7,24].

## 2. Materials and Methods

Case reports from 1963 until October 2024 have been selected from Pubmed and Scopus databases. Search terms included “eosinophilic pneumonia AND antidepressants”, as well as “eosinophilic pneumonia” and [name of specific antidepressant listed on Pneumotox]”, which has been listed as potentially causative of eosinophilic pneumonia on Pneumotox. Sources included in this review were selected based on mentioning eosinophilic pneumonia due to treatment with any antidepressant. Data extraction encompassed variables such as age, gender, implicated antidepressant and dosage, duration from medication initiation to symptom onset, symptomatology and progression, blood eosinophil counts, eosinophil fractions in bronchoalveolar lavage (BAL) fluid, initial radiographic findings from chest radiographs and computed tomography scans, and therapeutic interventions.

### 2.1. Inclusion and Exclusion Criteria

As eosinophilic pneumonia is only part of a small subset of eosinophilic lung diseases, a significant portion of the initially identified literature fell outside of our inclusion criteria. We included cases with acute or chronic eosinophilic pneumonia due to antidepressants. Patients with DRESS as a primary manifestation, drug-induced lupus, or other manifestations associated with eosinophilia were not included in the scoping review. Even though tryptophan or l-tryptophan has been studied to have positive effects on mood in healthy individuals [27] and the discussed role of tryptophan metabolism in depression disorder [28], l-tryptophan or tryptophan is not a recognized antidepressant. Therefore, despite its association with eosinophilia-myalgia syndrome [29], this review did not conduct further research into this substance.

### 2.2. Selection Process of Papers and Case Studies

Literature from 1963 until October 2024 has been selected from Pubmed and Scopus. Search terms included “eosinophilic pneumonia AND antidepressants”. Further research on those databanks included the search terms “eosinophilic pneumonia AND *the name of antidepressant listed on pneumotox.”* This list includes *amitriptyline, clomipramine, desipramine, duloxetine, fluoxetine, imipramine, maprotiline, metapramine, mianserin, nomifensine, sertraline, trazodone, trimipramine, venlafaxine, paroxetine, bupropion,* and *vortioxetine* based on the primary search on Pubmed and Scopus. Search terms were defined by two of the co-authors (F.D. and P.S.). Screening of the databases was performed by J.S. and L.S. Selection of the articles to be included was conducted by J.S., L.S., and F.D. Finally, another co-author (K.B.) independently checked the selected articles for relevance. This search resulted in 91 results that were then closely analyzed. Sources included in this review were selected based on mentioning eosinophilic pneumonia due to treatment with any type of antidepressant.

**Figure 1:**
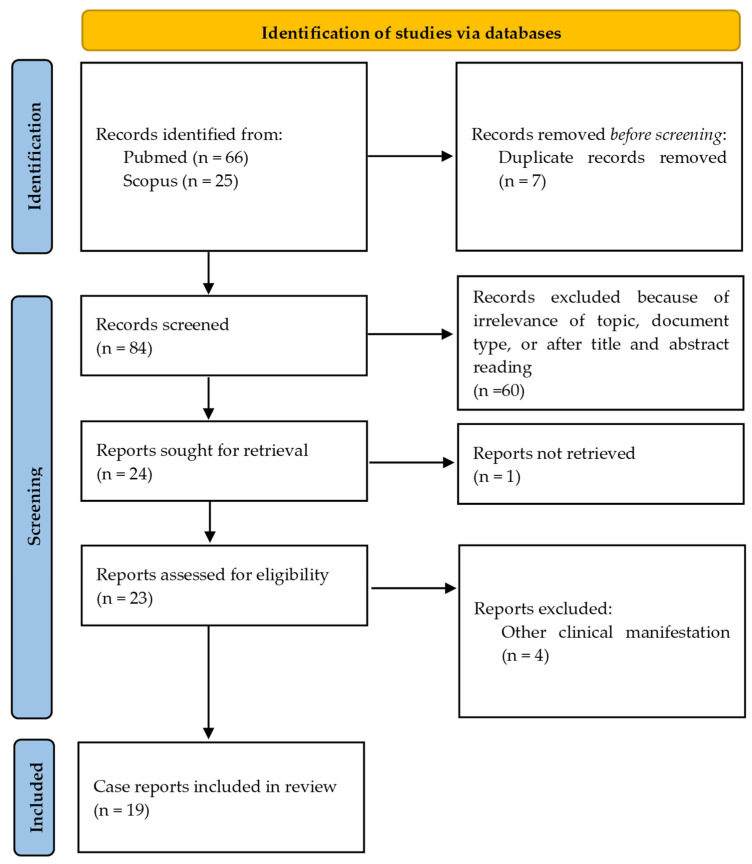
PRISMA flowchart of the search process.

The types of studies used in this review are case reports. Since a scoping review was performed, a PROSPERO registration was not completed. Publications in the English, French, or German language were reviewed; one resource had to be excluded based on language [30]. The search flow chart is shown in Figure 1.

## 3. Results

### 3.1. Presentation of the Included Studies

The presentation of the included case reports shows that eosinophilic pneumonia may be a rare side effect of antidepressants. Acute and chronic forms of eosinophilic pneumonia have been described in the literature and are associated with various classes of antidepressants. We identified nineteen case reports involving antidepressants. In one case, two suspect antidepressants were reported [31]. In another case, the patient presented with eosinophilic pneumonia, initially induced by sertraline [11]. After discontinuation of sertraline, venlafaxine was prescribed. However, the patient developed eosinophilic pneumonia due to venlafaxine [11]. In two cases, the patients presented with respiratory symptoms, pulmonary infiltrates, and peripheral eosinophilia after imipramine treatment [32,33]. The authors reported these cases as Löffler’s syndrome, a form of transient eosinophilic pulmonary infiltrate [32,33]. After carefully reviewing the two cases, we decided to include them in the analysis since Löffler’s syndrome is a form of eosinophilic pneumonia [2]. The medications associated with eosinophilic pneumonia are shown in Table 2.

### 3.2. Analysis

The clinical aspects of the case reports will be further discussed. A summary of the key clinical findings is presented in Table 3.

#### 3.2.1. Presentation of Patients

The case reports analyzed twelve women and seven men; the age range extended from 24 to 80 years. The median age of the patients was 42 years. A median age of approximately 40 years has been reported for patients with idiopathic CEP and 30 years for patients with idiopathic AEP [2].

#### 3.2.2. Patterns in Course of Symptoms

The onset of symptoms since drug initiation lay between 2 days and 10 months in the cases of eosinophilic pneumonia. Symptoms mainly occurred within the first weeks of treatment with the antidepressant in cases of AEP, while one study reported an interval of six months between drug initiation and AEP [10]. Cases that described a higher timeline window between the first intake of antidepressants and the onset of respiratory symptoms correlated with a recent drug dose increase (up to 4 weeks) before the diagnosis of AEP. Cases of CEP tended to show symptoms within the first months of drug intake. While some patients were initially hospitalized for psychiatric reasons, they described typical symptoms of EP, such as dry cough and dyspnea, during their hospital stay.

One case of EP was associated with a medical history of asthma [32], and only two case reports were associated with smoking [36,41]. However, some papers did not provide clinical information regarding smoking or asthma as associated conditions or risk factors for the presentation of eosinophilic pneumonia.

#### 3.2.3. Diagnostic Approaches

In the reviewed AEP and CEP case reports, various diagnostic approaches have been used to establish the diagnosis and elucidate the underlying cause. These approaches include radiological imaging, such as chest X-ray and CT scans, bronchoscopy with bronchoalveolar lavage (BAL), and lung biopsies.

Radiological imaging, chest X-rays, and computed tomography (CT) scans played a significant role in diagnosing AEP and CEP across all case reports. In one case, radiological findings were not reported [13]. The CT scans revealed bilaterally patchy ground glass opacities, primarily in the mid- and upper-lung zones. However, the lower lobes were also affected [36]. Regarding chest X-rays, bilateral alveolar infiltrates were observed, with a high localization variability.

A bronchoscopy with bronchoalveolar lavage (BAL) was of paramount importance for diagnosing AEP and CEP. Within the case reports, we observed an eosinophil variability from 25% to 80% for AEP, while the two cases of CEP reported a BAL eosinophil percentage of 15% and 18%. A percentage of BAL eosinophils above 25% is considered diagnostic of both AEP and CEP [2]. In our review, all AEP cases had a BAL eosinophilia above 25%. Although BAL eosinophils were below 25% in our CEP cases, the clinical and radiological findings were consistent with the disease.

#### 3.2.4. Treatment

Crucial to the treatment of these cases was the discontinuation of the responsible antidepressant. In addition, most cases report the use of corticosteroids to support patients in remission with respiratory failure. The primary medication for achieving a stable clinical course was the administration of different dosages of methylprednisolone and prednisolone over 3 to 7 days, on average. Next to corticosteroids, many patients have been first treated with empiric antibiotics with a presumptive diagnosis of bacterial pneumonia, without signs of clinical improvement.

#### 3.2.5. Clinical Outcome and Remission

A remarkable observation of all case reports is the absence of fatal outcomes, even in severe respiratory failure that led to patients’ intubation or ICU admission [10,36,37,39,41]. Another significant observation is that all patients manifested complete remission without relapse after a prolonged follow-up.

### 3.3. Limitations of Data Reported in the Clinical Cases

It is to be noted that some of the case reports did not lead to diagnosis using bronchoalveolar lavage (BAL) or lung biopsy. BAL is a criterion usually required for diagnosis because it is a minimally invasive method. One report justified not having fulfilled this requirement due to the unstable condition of the patient at the time of presentation [41], and another report described that the patient refused to undergo an endoscopic procedure [31].

## 4. Discussion

The reviewed case reports provide evidence of an association between antidepressant medications and the development of eosinophilic pneumonia. Different classes of antidepressants are involved. Acute and chronic forms of EP were reported in patients, mainly within a few weeks after the start of treatment with antidepressants. Although eosinophilic pneumonia is considered a hypersensitivity reaction, the dose of the responsible medication may play a causative role, since the disease occasionally manifests after dose step-up [9]. Additionally, a combination of antidepressant medications may lower the cut-off for the occurrence of eosinophilic pneumonia [31]. Furthermore, eosinophilic pneumonia may occur with more than one antidepressant in the same patient, as was the case with sertraline and venlafaxine when prescribed consecutively [11]. In some cases, the patients were already hospitalized due to their psychiatric illness [16,38]. In such patients, dyspnea may be underestimated or even attributed to the psychiatric disease, which makes the diagnosis of eosinophilic pneumonia more challenging [16,38]. Since eosinophilic pneumonia is rare, differential diagnosis of infiltrates in a chest X-ray includes respiratory infection. Eosinophilia in a differential blood count may be lacking, as is often the case in AEP [42]. Empiric antibiotic treatment was initiated in all patients, particularly those with AEP. A high degree of suspicion for other causes of pulmonary infiltrates is required when a patients’ response to antibiotics is not observed promptly. In such cases, aggressive diagnostic evaluation with computed tomography of the lungs [2] and bronchoscopy with BAL can secure the diagnosis [43]. Although, in our review, patients with CEP had an eosinophil BAL percentage below 25%, the clinical course, radiological findings, and BAL eosinophilia suggested an eosinophilic pneumonia [12,15]. Supportive treatment with oxygen and non-invasive and occasionally invasive ventilation is required in cases of respiratory failure [9,38,39], and until corticosteroids have led to remission. Discontinuation of the responsible drug is of paramount importance for disease resolution [5].

**Figure 2:**
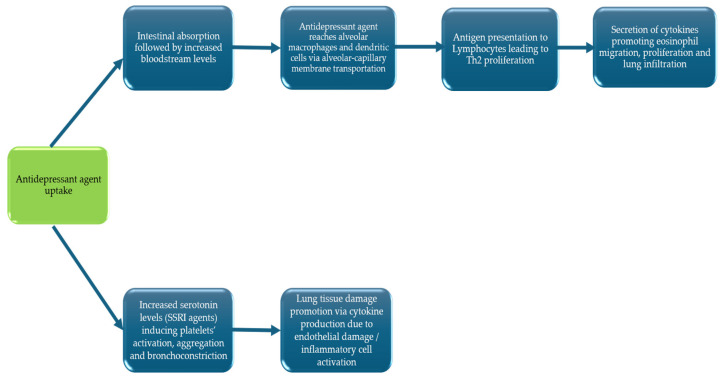
Putative pathogenetic mechanisms associated with antidepressant-induced eosinophilic pneumonia.

The pathophysiological mechanisms underlying EP induced by antidepressants remain largely unknown. However, research has shown serotonin’s central role as an immune modulator, with nearly all immune cells expressing at least one serotonergic receptor [44]. Drug-induced eosinophilic pneumonia may occur due to increased endothelial permeability induced by substances, such as serotonin [42]. Serotonin has been reported to regulate endothelial cells’ cytoskeleton and participate in their barrier function [45]. Serotonin may influence vascular endothelial cell inflammation, affecting the recruitment of eosinophils in the lungs [46,47]. Endothelial cells release IL-33, which is involved in eosinophil recruitment and degranulation [42]. Antidepressant medication, especially SSRIs, acts mainly by inhibiting the reabsorption of serotonin, increasing its concentration. Serotonin’s role as a smooth muscle constrictor is well known, along with the enhancement of platelet aggregation and activation [11]. Lung and alveoli damage is linked with the activation of platelets, neutrophils, and the secretion of cytokines that promote epithelial and endothelial damage, destruction of tight junctions, migration, and proliferation of inflammatory cells, as well as an influx of inflammatory exudate fluid inside alveoli [11]. Serotonin has been reported to lead to chemoattraction of eosinophils, similar to the action of eotaxin [48]. Endothelial injury leads to phagocytosis of drug components by alveolar macrophages, which play a significant role in the phagocytosis of toxic substances, drugs, and microorganisms [42]. It is hypothesized that antigen presentation from alveolar macrophages induces a Th2 proliferation of lymphocytes. Secretion of Th2 cytokines, such as IL-5, promote eosinophil and neutrophil migration and proliferation [42]. Dendritic cells may also contribute to this phenomenon. As mentioned in the pathogenesis, serotonin can influence the T-cell priming capacity of dendritic cells in favor of a Th2 response [21,42]. It is suggested that more contributing factors must be accumulated to result in eosinophil inflammation. Such factors may include specific gene polymorphisms, host innate and adaptive reactions, along with environmental factors like smoke and air pollutants [5]. A graphical hypothesis of the pathophysiological pathways, which may be involved in antidepressant-induced eosinophilic pneumonia, is shown in Figure 2.

## 5. Limitations of the Review

Given the rarity of eosinophilic pneumonia, there has been a scarcity of case reports published over the past few decades regarding its association with antidepressants, thereby constraining the breadth of the literature review. Additionally, the heterogeneous nature of the published case reports reduces the robustness of the data analysis. Different definitions and the lack of a complete diagnostic work-up, including CT of the lung and BAL, contribute to the heterogeneity of the reports analyzed.

## 6. Conclusions

In summary, the case reports reviewed emphasize the importance of considering antidepressants as potential triggers of eosinophilic pneumonia and highlight the need for greater awareness, early recognition, and appropriate management of this rare but severe adverse drug reaction in clinical practice.

## Figures and Tables

**Table 1:** Classification of Eosinophilic Pneumonias (modified from Refs. [1,2,3]).

**Eosinophilic Pneumonias of Known Cause:**
- Parasitic eosinophilic pneumonias
- Eosinophilic pneumonias of other infectious causes
- Drug-induced eosinophilic pneumonias (acute, chronic)
- Allergic bronchopulmonary aspergillosis and related syndromes
**Eosinophilic Pneumonias of Unknown Cause:**
*Idiopathic eosinophilic pneumonia:*
- Acute eosinophilic pneumonia
- Chronic eosinophilic pneumonia
*Eosinophilic Pneumonias Associated with Systemic Syndromes:*
- Eosinophilic Granulomatosis with Polyangiitis (EGPA, formerly known as Churg–Strauss Syndrome)
- Hypereosinophilic Syndrome
**Other Lung Diseases with Possible Eosinophilia:**
- Organizing pneumonia
- Bronchial asthma
- Desquamative interstitial pneumonia
- Langerhans cell histiocytosis
- Malignant diseases
- Sarcoidosis

**Table 2:** Presentation on antidepressants associated with eosinophilic pneumonia.

Antidepressant	Classification	Number of Cases
Sertraline	SSRI	5 [9,10,11,12,31]
Desipramine	SSRI	3 [13,34,35]
Venlafaxine	SNRI	3 [11,36,37]
Clomipramine	Tricyclic	2 [31,38]
Imipramine	Tricyclic	2 [32,33]
Trazodone	SARI	1 [39]
Duloxetine	SNRI	1 [15]
Trimipramine	Tricyclic	1 [16]
Amitriptyline	Tricyclic	1 [40]
Vortioxetine	SMS	1 [41]
Maprotiline	Tetracyclic	1 [14]

**Table 3:** Overview of clinical findings and outcomes of patients with eosinophilic pneumonia due to antidepressants.

References	Medication	Lung Involvement	Dosage of Drug	Duration to Symptom Onset	Patient Age	Sex	History of Smoking	Asthma	BAL Eos	CXR/CT	Treatment Dosage	Treatment Duration	ICU/Intubation	Oxygen Therapy	Outcome
Brancaleone P. et al. [12]	Sertraline	CEP*	100 mg	10 months	69	F	no	n/m	18%	yes	Sertraline cessation	-	-	-	Remission
Adhikari P. et al. [9]	Sertraline	AEP*	200 mg	2 months	76	F	no	no	25%	yes	MP (3 gr) to PD (60 mg)	n/m	yes/no	yes	Remission
Muftah M. et al. [10]	Sertraline	AEP	100 mg	6 months	49	F	no	n/m	41%	yes	PD 40 mg	3 days	no/no	yes	Remission
Tsigkaropoulou et al. [11]	Sertraline	AEP	-	-	80	F	no	n/m	n/m	n/m	n/m	7+ days	no/no	n/m	Remission
Barnes M.T. et al. [31]	Sertraline Clomipramine	AEP	50 mg 50 mg	1 week 4 weeks	40	F	no	no	n/p	yes	SSRI cessation	-	no/no	n/m	Remission
Cimen P. et al. [38]	Clomipramine	AEP	75 mg	6 days	38	F	yes	no	30%	yes	MP 60 mg	4 weeks	yes/no	NIV	Remission
Panuska J.R. et al. [13]	Desipramine	CEP	200 mg	3 weeks	52	M	no	no	n/m	yes	Aminophylline/ inhaled β2 agonist	n/m	no/no	n/m	Remission
Kraus R.P. et al. [34]	Desipramine	AEP	150 mg	13 days	67	F	no	no	n/m	yes	Desipramine cessation	-	no/no	yes	Remission
Mutnick A. et al. [35]	Desipramine	AEP	150 mg	7 days	39	F	no	no	n/p	yes	MP 60 mg PD 20 mg	n/m	no/no	yes	Remission
Tsigkaropoulou et al. [11]	Venlafaxine	AEP	225 mg	1 month	80	F	no	n/m	28%	yes	PD 10 mg	n/m	no/no	yes	Remission
Paparrigopoulos et al. [37]	Venlafaxine	AEP	6.3 g overdose	2 days	27	M	no	no	80%	yes	MP (1.8 g) to PD (60 mg)	3 months	yes/yes	yes	Remission
Fleisch M.C. et al. [36]	Venlafaxine	AEP	75 mg	2 weeks	41	M	yes	no	25%	yes	MP 3 g	3 days	yes/no	yes	Remission
Wilson I.C et al. [33]	Imipramine	Löffler’s syndrome	300 mg	11 days	43	F	n/m	n/m	n/p	yes	Imipramine cessation	-	no/no	no	Remission
Amsterdam J.D. [32]	Imipramine	Löffler’s syndrome	300 mg	30 days	36	F	n/m	yes	n/p	yes	Imipramine cessation	-	no/no	no	Remission
Gaudenz R. et al.≠ [14]	Maprotiline	CEP		3 months	74	M				yes	Maprotiline cessation				Remission
Espeleta V.J. et al. [15]	Duloxetine	CEP	-	4 months	32	M	no	no	15%	yes	Duloxetine cessation	-	no/no	no	Remission
Salerno S.M. et al. [39]	Trazodone	AEP	1.5 g overdose	5 days	31	F	no	no	80%	yes	MP (dose n/m)	7 days	yes/yes	yes	Remission
Noh H. et al. [40]	Amitriptyline	AEP	5 mg	8 days	26	F	n/m	no	38%	yes	Amitriptyline cessation	-	no/no	n/m	Remission
Eigenmann A.K. et al. [16]	Trimipramine	CEP	300 mg	5 weeks	55	M	no	no	n/m	yes	PD 50 mg	n/m	no/no	n/m	Remission
O’Brien T.M. et al. [41]	Vortioxetine	AEP	10 mg	3 weeks	35	M	n/m	no	n/p	yes	MP—500 mg	2 days	Yes/no	yes	Remission

CEP = Chronic Eosinophilic Pneumonia, AEP = Acute Eosinophilic Pneumonia, n/m = not mentioned, DRESS = Drug Reaction with Eosinophilia and Systematic Symptoms, MP = Methylprednisolone, NIV: non-invasive ventilation, PD = Prednisolone, n/m = not mentioned, n/p = not performed, SSRI = Selective Serotonin Reuptake Inhibitor, ≠: only abstract available.
